# School Health Needs Assessment in Chanchamayo, Peru: A Health Promoting School Project

**DOI:** 10.3389/fpubh.2020.00333

**Published:** 2020-07-21

**Authors:** Yoona Choi, Sunjoo Kang, Jin Sun Kim, Insook Kwon, Myungken Lee

**Affiliations:** ^1^Department of Nursing, Ulsan College, Ulsan, South Korea; ^2^Department of Global Health, Graduate School of Public Health, Yonsei University, Seoul, South Korea; ^3^Department of Nursing, Chosun University, Gwangju, South Korea; ^4^Ewha Womans High School, Seoul, South Korea

**Keywords:** children, adolescent, needs assessment, school health, Peru

## Abstract

**Background:** School-based health promotion can be particularly valuable in developing countries. However, there is a lack of information about the health needs of Peruvian school students. The purpose of this study was to conduct a health needs assessment to develop strategies for a school health promotion program in a jungle and indigent region in Chanchamayo, Peru.

**Methods:** This study was conducted using a mixed method approach that included a literature review, national and local statistics, stakeholder interviews, and a survey. Participants of the survey were 210 teachers, 2,504 elementary school students, and 2,834 secondary school students from six ‘schools in two planned project implementation regions. A self-administered questionnaire for students was developed based on WHO's Global School-based Student Health Survey. Collected data were analyzed using descriptive statistics, chi-square tests, and *t*-tests for the survey data and content analysis for the interviews.

**Results:** Weak school health systems were identified, including school health policies, curriculum, trained health care personnel, and health-related facilities and equipment. Common health problems of students were anemia, nutritional deficiency, infectious diseases, tuberculosis, drug abuse, poor hygiene, and sex-related problems. High absence rates from school due to family problems and high dropout rates due to pregnancy were also critical issues. Teachers identified personal hygiene, nutrition, reproductive health, and sex education as high priorities for school health education, while students identified prevention of infectious diseases, nutrition education, psychological health, and healthy lifestyles as priorities. Identified strategies included: establishment of school health policies, curriculum-based interventions, increasing community participation and raising school health awareness, capacity building for health care promotors, training of trainers, and partnership between schools and communities.

**Conclusions:** Findings from this study will help guide the development and implementation of a school-based health promotion program in Chanchamayo. Multicomponent school-based interventions that consider feasibility and sustainability will be developed and evaluated based on WHO's Health Promoting School concepts.

## Introduction

School health is an essential element for achieving education goals by maintaining health and promoting students and staff in schools (1). Students require physical and mental support for development, so it is vital to promote their health through the school system in which students spend most of their day. In addition, school health also has the effect of reducing the consequences of health inequality that result in poor health (2) while improving students' health behaviors, health literacy, and academic achievements (3). Two factors should highlight school health: the practical targets for children, adolescents, and community access and the integrated school health program. Schools should provide disease-prevention interventions with an interest in the health problems of students and their families and provide a variety of integrated health education and activities (4). Thus, school health, which determines a healthy future through education, has emerged as a need requiring long-term projects to promote school health. School-based health promotion projects are especially beneficial in developing countries that are suffering from limited health literacy and a high burden of diseases (3).

Health promoting schools (HPS) developed based on the relationship between health and education by the World Health Organization (WHO) in the late 1980s (2). Components of HPS include “school health policies, the physical environment of school, school's social environment, community relationships, personal health skills, and health services” (5). Whitman (6) suggested the components of HPS based on the WHO framework are school health policies, school health service, school health education, school environment, and community networks. HPS are important in achieving both health and academic goals through prevention of communicable diseases and unhealthy behaviors (1). HPS, where all members of the school community participate in improving health, need a comprehensive approach, rather than only one or two programs (7).

Peru is an upper-middle income country that has recently experienced economic growth. However, severe social and regional disparities arise because rural areas have difficulties gaining access to cities near the Andes and the Amazon (8). In rural areas of Peru, health inequalities are severe, with a poverty rate that is three times higher, an infant mortality two times higher, and a chronic malnutrition three times higher than in urban areas (9). In addition, the adolescent birth rate in rural areas (110 births per 1,000) was higher than in urban areas (58 births per 1,000), according to the Pan American Health Organization (9). Over 90% of children of primary school age and over 80% of children of lower secondary school age were enrolled in school globally in 2019 (10). However, only 80% of the school age population aged 10–19 in Peru were enrolled in school. Moreover, the prevalence of drinking (36.0%), smoking (21.0%), and cocaine use (0.4%) was reported to be high among the school age population in Peru (9). The Global School Health Survey (GSHS) of Peru reported 19.7% of adolescents aged 13–17 had sexual intercourse and only 34% of girls between 15 and 24 years old had knowledge of condom use (11). Health conditions for those of school age are closely related to health conditions in adulthood, and the lifestyle habits formed during this period are continued until adulthood. Therefore, it is essential to improve health for the school age population (12). Thus, programs for school-age children and adolescents in rural areas should focus on promoting health using school-based health promotion.

Health needs assessment guides the procedure to plan and implement health activities based on the health and healthcare needs of a specific group (13). When identifying the appropriate health needs of a community, priorities of community health program can be established, current resources can be identified, and community participants can become involved (14). However, there is a lack of information about the health needs of Peruvian school students. Therefore, the health and education sectors in developing countries need to assess the health needs of their school-age children and adolescents to adapt the school health promotion programs for enhancing their overall well-being. The purpose of this study was to conduct a health needs assessment to develop strategies for an HPS project in a jungle and indigent region in Chanchamayo, Peru.

## Materials and Methods

### Study Design

This study was conducted using a mixed method to understand the needs of school health in rural areas in Peru. The quantitative study involved administering structured questionnaires to students and teachers, while the qualitative study included conducting semi-structured interviews with 15 key informants.

The five components of HPS including school health policies, school health service, school health education, school environment, and community networks (6) were used as a framework to assess health needs in school for both quantitative and qualitative data collection. The project team leading the school health needs assessment and representatives from the Ministry of Health, regional health directorate, provincial government, Chanchamayo City Hall, and regional health center were involved to advise on the project.

### Study Sampling

The Perené and Pichanaqui districts in Chanchamayo, where the project of “Public Health Capacity-building project in Chanchamayo, Peru” was initiated by the Korea International Cooperation Agency (KOICA), which was the funding agency, were selected as the two target areas. The people living in the Perené district are more vulnerable to poorer health conditions compared to other regions due to a lack of infrastructure for health care facilities, medical services, schools, and jobs related to unplanned population growth (15). In addition, a high distribution of Aboriginal people live in the forests near Perené (15, 16). This region still has a high incidence of malaria and dengue, and tropical infectious and parasitic diseases remain unsolved health problems (16). Pichanaqui is located next to Perené and one health center in Pichanaqui managed both the Perené and Pichanaqui districts (15).

**Figure 1:**
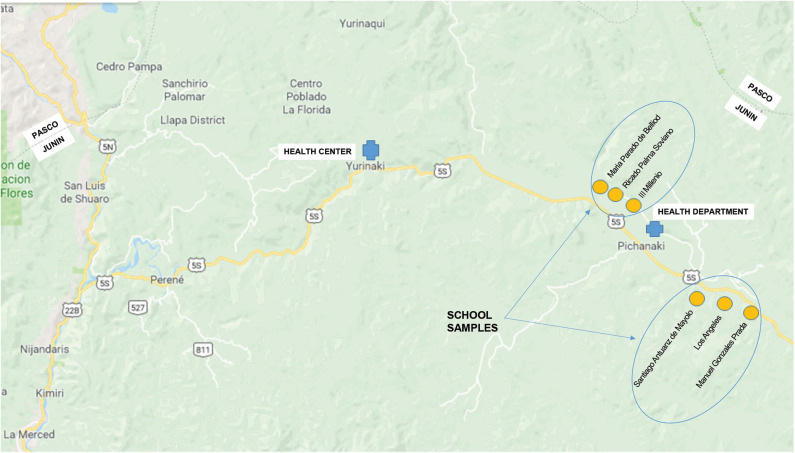
Map of the study area.

Six public schools that were located in a populated area were selected for this study (Figure 1). First, public schools among the regional schools were selected using a total list of schools. Second, the schools that operated both primary and secondary schools were selected. Third, the schools with more than 400 students were selected. Finally, in order to compare the regions between the two selected districts, three schools that had good cooperation with the project in the two districts, respectively, were selected. The final target population was 310 teachers, 3,568 primary school students, and 3,736 secondary school students from the six selected schools. Participants who refused to participate (100 teachers, 1,064 primary school students, and 902 secondary school students) were excluded. As a result, 210 teachers, 2,504 primary school students, and 2,834 secondary school students from six schools participated in the survey.

Regarding key person interviews, the participants included principals of the six selected schools, teachers who were in charge of students' health, and health professionals including a doctor, two nurses, and a health promotor of health center in Perené.

### Survey Instrument

The survey questionnaire was developed using a self-administered structured questionnaire based on the WHO Global School-based Student Health Survey Questionnaire (17). Moreover, this instrument was reviewed by the Ministry of Health of Peru and regional health professionals for content validity with the Spanish version and modified to use in this study. The questionnaire for teachers was composed of general characteristics, health problems, health behaviors, and the needs of health education for students which comprised of 44 questions. The number of questions for primary school students and secondary school students were 43 and 57 items, respectively. The questionnaire for students included general characteristics, family health problems, health behaviors, personal relationships, health knowledge, family environment, and health education needs. HIV-related knowledge for secondary school students were given a score of 0 or 1 (0 = wrong answer, 1 = correct answer) among six questions.

The semi-structured interview questions for key informants were developed based on components of HPS and two experts' review of content validity (6). In this study two Chanchamayo health sector experts were requested to evaluate content validity, after which appropriate which modifications were made. In terms of the key person interviews, the questions included the priority of health problems in the areas, major health problems among students and teachers, current school health policies, school health services, school health facilities, school health education, and school health environment such as physical safety, nutrition in the school snack bar, and networking with the community.

### Data Acquisition

Representative schoolteachers from six schools were trained on how to conduct the 2 h survey by the Korean nursing faculty from the project team using the written manual, and parents of children in lower levels of primary schools also helped their children to conduct the survey. Informed consent was obtained from each school and from all participants' parents or guardians in advance, and an anonymous questionnaire was used. School health needs assessment was conducted from July to October 2014. Descriptive statistics, chi-square tests, and *t*-tests were used to analyze the general and health-related characteristics and health education program needs.

The semi-structured interviews were conducted from July 14 to 25, 2014. The interviews were conducted at the offices or conference rooms of 15 key informants; one manager of school health in the provincial education department, two directors of the regional health center, 12 school principals and teachers from six schools.

### Data Analysis

All quantitative data were analyzed using descriptive statistics including real number, percentages, means, and standard deviations. The interview data were analyzed using an inductive approach by thematic content analysis.

### Ethical Approval

The school health needs assessment including a survey and a key person interview received approval from the Regional Health Directorate in Peru and the Korea International Cooperation Agency. In addition, research ethics approval was obtained from the Institutional Review Board of Chosun University (IRB No. 2-1041055-AB-N-01-2019-3) to use school health needs assessment data for secondary data analysis.

## Results

### General and Health-Related Characteristics of Schoolteachers

More than sixty percent of the participants were female (62.4%). In terms of age, 44.8% were 40–49 years old, 30.0% were 30–39 years old, and 20.5% were more than 50 years old (Mean ± SD = 43.1 ± 8.24).

Most of the teachers graduated from a university (91.9%) and almost 40% of participants had more than 20 years working experience as schoolteachers. Regarding health status, most of the participants reported their health status was fair (58.1%) or good (39.5%). Based on the body mass index, 45.7% were overweight, and 9.5% were obese. Thirty percent of participants reported that they did not exercise and 50.5% did exercise one or two times per week. Most of participants reported they were non-smokers (99.0%) and non-drinkers (79.5%). About 28.0% had a moderate level of stress and 20.0% had a high level of stress. In terms of receiving health education in last year, more than half participated in health education (60.5%) and reproductive health education (51.9%); however, only 39.5% were educated in first aid. Schoolteachers from Pichanaqui were receiving significantly more health education and reproductive health education (72.6 and 57.0%, respectively) than schoolteachers from Perené (38.7 and 42.7%) (χ^2^ = 23.22, *p* < 0.001; χ^2^ = 3.99, *p* = 0.046).

The mean health knowledge was 2.3 (± 0.47) out of 3. Regarding the needs of health education for students, the highest priority was personal hygiene (46.7%), followed by sexual and reproductive health (24.8%), nutrition (16.2%), and healthy behaviors (6.7%) (Table 1).

**Table 1:** General and health related characteristics of schoolteachers by regions (*N* = 210).

**Characteristics**	**Category**	***n*** **(%)/mean (±SD)**			**χ ^**2**^ (*p*)/t (*p*)**
		**Total**	**Perené**	**Pichanaqui**	
Gender	Male	79 (37.6)	30 (40.0)	49 (36.3)	0.28 (0.596)
	Female	131 (62.4)	45 (60.0)	86 (63.7)	
Age	<30 years old	10 (4.8)	4 (5.3)	6 (4.4)	1.53 (0.675)
	30~ <40 years old	63 (30.0)	26 (34.7)	37 (27.4)	
	40~ <50 years old	94 (44.8)	30 (40.0)	64 (47.4)	
	≥50 years old	43 (20.5)	15 (20.0)	28 (20.7)	
Highest education level	High school	6 (2.9)	3 (4.0)	3 (2.2)	1.62 (0.445)
	College	10 (4.8)	2 (2.7)	8 (5.9)	
	University	193 (91.9)	70 (93.3)	124 (91.9)	
Working period	<10 years	45 (21.4)	17 (22.7)	28 (20.7)	6.73 (0.081)
	10~ <15 years	34 (16.2)	18 (24.0)	16 (11.9)	
	15~ <20 years	50 (23.8)	13 (17.3)	37 (27.4)	
	≥20 years	81 (38.6)	27 (36.0)	54 (40.0)	
Health status	Good	83 (39.5)	1 (1.3)	4 (3.0)	0.92 (0.632)
	Fair	122 (58.1)	42 (56.0)	80 (59.3)	
	Poor	5 (2.4)	32 (42.7)	51 (37.8)	
Body Mass Index	Under-weight	1 (0.5)	1 (1.3)	0 (0.0)	5.90 (0.117)
	Normal	93 (44.3)	34 (45.3)	59 (43.7)	
	Over-weight	96 (45.7)	37 (49.3)	59 (43.7)	
	Obese	20 (9.5)	3 (4.0)	17 (12.6)	
Exercise	None	63 (30.0)	16 (21.3)	47 (34.8)	4.52 (0.104)
	1–2 times/week	106 (50.5)	41 (54.7)	65 (48.1)	
	More than 3 times/week	41 (19.5)	18 (24.0)	23 (17.0)	
Smoking	Yes	2 (1.0)	1 (1.3)	1 (0.7)	0.18 (0.672)
	No	208 (99.0)	74 (98.7)	134 (99.3)	
Drinking	Yes	43 (20.5)	15 (20.0_)	28 (20.7)	0.02 (0.899)
	No	167 (79.5)	60 (80.0)	107 (79.3)	
Stress	No	19 (9.0)	8 (10.7)	11 (8.1)	3.39 (0.335)
	Low	90 (42.9)	35 (46.7)	55 (40.7)	
	Moderate	59 (28.1)	22 (29.3)	37 (27.4)	
	High	42 (20.0)	10 (13.3)	32 (23.7)	
Receiving health education in the last year	Yes	127 (60.5)	29 (38.7)	98 (72.6)	23.22 (< 0.001)
	No	83 (39.5)	46 (61.3)	37 (27.4)	
Receiving first aid education in the last year	Yes	67 (31.9)	22 (29.3)	45 (33.3)	0.36 (0.551)
	No	143 (68.1)	53 (70.7)	90 (66.7)	
Receiving reproductive health education in the last year	Yes	109 (51.9)	32 (42.7)	77 (57.0)	3.99 (0.046)
	No	101 (48.1)	43 (57.3)	58 (43.0)	
Health knowledge (3 points)		2.3 (± 0.47)	2.29 (± 0.50)	2.36 (± 0.45)	−0.98 (0.331)
Needs of health education for students	Personal hygiene	98 (46.7)	33 (44.0)	65 (48.1)	4.20 (0.522)
	Sexual & reproductive health	52 (24.8)	21 (28.0)	31 (23.0)	
	Nutrition	34 (16.2)	12 (16.0)	10 (7.4)	
	Healthy behaviors	14 (6.7)	4 (5.3)	22 (16.3)	
	Mental health	9 (4.3)	5 (6.7)	4 (3.0)	
	Prevention of communicable diseases	3 (1.4)	0 (0.0)	3 (2.2)	

### General and Health-Related Characteristics of Primary Students

Slightly more than half of the participants were male (50.6%) and the family social economic status of most students was at a middle level (65.9%). More students from Perené reported that their family social economic status was a low level (33.2%) compared to the students from Pichanaqui (22.7%) (χ^2^ = 31.14, *p* < 0.001). More than half of the students had good health status (59.3%), and more students from Perené (64.2%) answered their health status was good compared to students from Pichanaqui (56.9%) (χ^2^ = 29.70, *p* < 0.001). Around 26% of students were either overweight or obese and there was a significant difference between students of Perené and Pichanaqui (χ^2^ = 15.67, *p* = 0.001). In terms of accidents near schools, the Perené area (7%) had more traffic accidents than Pichanaqui (4.9%) (χ^2^ = 13.88, *p* = 0.008). A quarter of students used the internet and had suffered from being bullied in school. Most of students reported that they washed their hands before having a meal or after going to the toilet; however, more students from Pichanaqui (34.8%) always washed their hands in those times compared to the students of Perené (22.9%) (χ^2^ = 51.77, *p* < 0.001). Regarding diet, about 15% of students always ate snacks and more students from Perené (12.5%) answered that they always ate snacks than students from Pichanaqui (6.6%) (χ^2^ = 54.95, *p* < 0.001). Likewise, 17.3% of students from Perené and 11.3% of students from Pichanaqui reported that they drank more than two bottles of soda per day (χ^2^ = 45.66, *p* < 0.001). Less than half of the students were educated in health education, while most of the students (87.6%) received personal hygiene education. In terms of knowledge about tooth brushing, 40.0% responded with correct answers. Almost a quarter of students reported their family relationships were good (72.2%) (Table 2).

**Table 2:** General and health related characteristics of primary school students by Regions (*N* = 2,504).

**Characteristics**	**Category**	***n*** **(%)**			**χ ^**2**^ (*p*)**
		**Total**	**Perené**	**Pichanaqui**	
Gender	Male	1,268 (50.6)	433 (52.5)	835 (49.7)	1.68 (0.195)
	Female	1,236 (49.4)	392 (47.5)	844 (50.3)	
Grade	1	232 (9.3)	112 (13.6)	120 (7.1)	41.10 (<0.001)
	2	450 (18.0)	136 (16.5)	314 (18.7)	
	3	408 (16.3)	100 (12.1)	308 (18.3)	
	4	446 (17.8)	152 (18.4)	294 (17.5)	
	5	427 (17.1)	135 (16.4)	282 (17.4)	
	6	541 (21.6)	190 (23.0)	351 (20.9)	
Social economic status	Low	655 (26.2)	274 (33.2)	381 (22.7)	31.14 (<0.001)
	Middle	1,651 (65.9)	491 (59.5)	1,161 (69.2)	
	High	197 (7.9)	60 (7.3)	137 (8.2)	
Health status	Poor	201 (8.0)	83 (10.1)	118 (7.0)	29.70 (<0.001)
	Fair	818 (32.7)	212 (25.7)	606 (36.1)	
	Good	1,485 (59.3)	530 (64.2)	955 (56.9)	
Body Mass Index	Under-weight	153 (6.1)	48 (5.8)	105 (6.3)	15.67 (0.001)
	Normal	1,697 (67.8)	601 (72.8)	1,096 (65.3)	
	Over-weight	457 (18.3)	124 (15.0)	333 (19.8)	
	Obese	197 (7.9)	52 (6.3)	145 (8.6)	
Type of accident nearby school	No accident	1,892 (75.6)	630 (76.4)	1,262 (75.2)	13.88 (0.008)
	Traffic accident	145 (5.8)	62 (7.5)	83 (4.9)	
	Slight accident	289 (11.5)	75 (9.1)	214 (12.7)	
	Severe accident	71 (2.8)	20 (2.4)	51 (3.0)	
	Other accident	107 (4.3)	38 (4.6)	69 (4.1)	
Using Internet	Yes	624 (24.9)	221 (26.8)	403 (24.0)	2.30 (0.130)
	No	1,880 (75.1)	604 (73.2)	1,276 (76.0)	
Experience of bullying in school	No	1,628 (65.0)	545 (66.1)	1,083 (64.5)	1.24 (0.745)
	Nearly not	270 (10.8)	89 (10.8)	181 (10.8)	
	Sometimes	489 (19.5)	151 (18.3)	338 (20.1)	
	Often & Always	117 (4.7)	40 (4.8)	77 (4.6)	
Experience of violence in school	No	1,685 (67.3)	579 (70.2)	1,106 (65.9)	6.60 (0.086)
	Nearly not	259 (10.3)	74 (9.0)	185 (11.0)	
	Sometimes	454 (18.1)	134 (16.2)	320 (19.1)	
	Often & Always	106 (4.2)	38 (4.6)	68 (4.1)	
Experience of violence at home	No	1,757 (70.2)	599 (72.6)	1,158 (69.0)	12.43 (0.006)
	Nearly not	262 (10.5)	67 (8.1)	195 (11.6)	
	Sometimes	423 (16.9)	131 (15.9)	292 (17.4)	
	Often & Always	62 (2.5)	28 (3.4)	34 (2.0)	
Hand washing before having meal or after restroom	No	73 (2.9)	38 (4.6)	35 (2.1)	51.77 (<0.001)
	Rarely	53 (2.1)	26 (3.2)	27 (1.6)	
	Sometimes	618 (24.7)	236 (28.6)	382 (22.8)	
	Often	987 (39.4)	336 (40.7)	651 (38.8)	
	always	773 (30.9)	189 (22.9)	584 (34.8)	
Tooth brushing per day	Never	52 (2.1)	16 (1.9)	36 (2.1)	8.41 (0.038)
	Once	280 (11.2)	111 (13.5)	169 (10.1)	
	Twice	1,028 (41.1)	315 (38.2)	713 (42.5)	
	More than 3 times	1,144 (45.6)	383 (46.4)	761 (45.3)	
Vegetable intake	Never	66 (2.6)	22 (2.7)	44 (2.6)	12.00 (0.017)
	Rarely	132 (5.3)	46 (5.6)	86 (5.1)	
	Sometimes	1,261 (50.4)	450 (54.5)	811 (48.3)	
	Often	484 (19.3)	133 (16.1)	351 (20.9)	
	always	561 (22.4)	174 (21.1)	387 (23.0)	
Snack intake	Never	196 (7.8)	53 (6.4)	143 (8.5)	54.95 (<0.001)
	Rarely	383 (15.3)	109 (13.2)	274 (16.3)	
	Sometimes	1,553 (62.1)	480 (58.2)	1,074 (64.0)	
	Often	157 (6.3)	80 (9.7)	77 (4.6)	
	always	214 (8.5)	103 (12.5)	111 (6.6)	
Soda intake per day	Never	608 (24.3)	139 (16.8)	469 (27.9)	45.66 (<0.001)
	< 1 bottle	827 (33.0)	282 (34.2)	545 (32.5)	
	1 bottle	736 (29.4)	261 (31.6)	475 (28.3)	
	More than 2 bottles	333 (13.3)	143 (17.3)	190 (11.3)	
Receiving personal hygiene education in last 1 year	Yes	2,193 (87.6)	697 (84.5)	1,496 (89.1)	10.84 (0.001)
	No	311 (12.4)	128 (15.5)	183 (10.9)	
Receiving health education in last 1 year	Yes	1,192 (47.6)	384 (46.5)	808 (48.1)	0.55 (0.457)
	No	1,312 (52.4)	441 (53.5)	871 (51.9)	
Knowledge of tooth brushing	Yes	1,001 (40.0)	321 (38.9)	680 (40.5)	0.58 (0.445)
	No	1,503 (60.0)	504 (61.1)	999 (59.5)	
Checking studying or homework from parents	Never	175 (7.0)	64 (7.8)	111 (6.6)	3.63 (0.459)
	Nearly not	130 (5.2)	37 (4.5)	93 (5.5)	
	Sometimes	1,339 (53.5)	449 (54.4)	890 (53.0)	
	Often	214 (8.5)	63 (7.6)	151 (9.0)	
	always	646 (25.8)	212 (25.7)	434 (25.8)	
Relationship with family	Poor	157 (6.3)	67 (8.1)	90 (5.4)	9.00 (0.011)
	Moderate	538 (21.5)	161 (19.5)	377 (22.5)	
	Good	1,809 (72.2)	597 (72.4)	1,212 (72.2)	

### General and Health-Related Characteristics of Secondary Students

More than half of the participants were female (53.1%) and most students reported their family social economic status was at a middle level (82.2%). Regarding health status, only 6.4% said their health status was poor. Based on body mass index, 13.9% of the students were either overweight or obese. More than half of the students used the Internet (60.0%) and student responses from the Pichanaqui area (64.3%) indicated more Internet usage than the Perené area (49.1%) (χ^2^ = 54.91, *p* < 0.001). Only 3.5% of students reported they were often or always bullied in school and 14.9% sometimes had an experience of bullying in school. Among secondary school students, 5.5% were smokers, 7.8% drank alcohol, and 9.4% had a sexual intercourse experience. The students from the Perené area were more likely to drink alcohol (9.6%) than those from the Pichanaqui area (7.1%) (χ^2^ = 5.04, *p* = 0.025) and the students from Perené (13.5%) had significantly more experience of sexual intercourse than the students from Pichanaqui (7.8%) (χ^2^ = 22.21, *p* < 0.001). Among the students who had sexual intercourse, the rate of condom use, and contraceptive method use at their last sexual intercourse were 66.7 and 53.2%, respectively.

In terms of health education, the students from Perené areas received less reproductive health education (62.0%) (χ^2^ = 79.81, *p* = < 0.001), HIV/AIDS education (79.5%) (χ^2^ = 38.05, *p* = < 0.001), and contraception education (60.3%) (χ^2^ = 10.76, *p* = 0.001) than the students from Pichanaqui (78.4, 88.5, and 66.8%, respectively). In addition, regarding knowledge of contraception and pregnancy, more students from Perené reported that they had no knowledge of them (44.5% and 42.9%) than the students from Pichanaqui (28.5 and 41.9%) (χ^2^ = 7.53, *p* = 0.023 and χ^2^ = 15.32, *p* < 0.001). However, regarding knowledge about HIV/AIDS, the students from Perené (4.06 ± 1.43 out of 6) had significantly higher knowledge than the students from Pichanaqui (3.91 ± 1.39) (*t* = 2.41, *p* = 0.016). The highest priority of health education needs was personal hygiene (34.0%), followed by sexual and reproductive health (30.2%) and healthy behaviors (10.9%) (Table 3).

**Table 3:** General and health related characteristics of secondary school students by Regions (*N* = 2,834).

**Characteristics**	**Category**	***n*** **(%)/mean (±** **SD)**			**χ^**2**^ (*p*)/t (*p*)**
		**Total**	**Perené**	**Pichanaqui**	
Gender	Male	1,328 (46.9)	379 (47.0)	949 (46.8)	0.01 (0.913)
	Female	1,506 (53.1)	427 (53.0)	1,079 (53.2)	
Grade	7	626 (22.1)	197 (24.4)	626 (22.1)	23.31 (<0.001)
	8	662 (23.4)	207 (25.7)	662 (23.4)	
	9	506 (17.9)	162 (20.1)	506 (17.9)	
	10	539 (19.0)	124 (15.4)	539 (19.0)	
	11	501 (17.7)	116 (14.4)	501 (17.7)	
Social economic status	Low	357 (12.6)	113 (14.0)	244 (12.0)	2.20 (0.334)
	Middle	2,320 (82.2)	650 (80.6)	1,680 (82.8)	
	High	147 (5.2)	43 (5.3)	104 (5.1)	
Health status	Poor	178 (6.3)	67 (8.3)	111 (5.5)	12.15 (0.002)
	Fair	912 (32.2)	231 (28.7)	681 (33.6)	
	Good	1,744 (61.5)	508 (63.0)	1,236 (60.9)	
Body Mass Index	Under-weight	174 (6.1)	63 (7.8)	111 (5.5)	6.46 (0.091)
	Normal	2,267 (80.0)	641 (79.5)	1,626 (80.2)	
	Over-weight	320 (11.3)	84 (10.4)	236 (11.6)	
	Obese	73 (2.6)	18 (2.2)	55 (2.7)	
Using Internet	Yes	1,699 (60.0)	396 (49.1)	1,303 (64.3)	54.91 (<0.001)
	No	1,135 (40.0)	410 (50.9)	725 (35.7)	
Experience of bullying in school	No	1,746 (61.6)	511 (63.4)	1,235 (60.9)	2.78 (0.427)
	Nearly not	566 (20.0)	155 (19.2)	411 (20.3)	
	Sometimes	423 (14.9)	109 (13.5)	314 (15.5)	
	Often & Always	99 (3.5)	31 (3.8)	68 (3.4)	
Experience of violence in school	No	1,715 (60.5)	539 (66.9)	1,176 (58.0)	19.4 (<0.001)
	Nearly not	506 (17.9)	121 (15.0)	385 (19.0)	
	Sometimes	502 (17.7)	117 (14.5)	385 (19.0)	
	Often & Always	111 (3.9)	29 (3.6)	82 (4.0)	
Experience of violence at home	No	2,317 (81.8)	659 (81.8)	1,658 (81.8)	2.36 (0.502)
	Nearly not	302 (10.7)	93 (11.5)	209 (10.3)	
	Sometimes	181 (6.4)	44 (5.5)	137 (6.8)	
	Often & Always	34 (1.2)	10 (1.2)	24 (1.2)	
Smoking	Yes	155 (5.5)	51 (6.3)	104 (5.1)	1.61 (0.205)
	No	2,679 (94.5)	755 (93.7)	1,924 (94.9)	
Drinking	Yes	220 (7.8)	77 (9.6)	143 (7.1)	5.04 (0.025)
	No	2,614 (92.2)	729 (90.4)	1,885 (92.9)	
Experience of sexual intercourse	Yes	267 (9.4)	109 (13.5)	158 (7.8)	22.21 (<0.001)
	No	2,567 (90.6)	697 (86.5)	1,870 (92.2)	
Using condom at last sexual intercourse	Yes	178 (66.7)	74 (67.9)	104 (65.8)	0.12 (0.725)
	No	89 (33.3)	35 (32.1)	54 (34.2)	
Using contraceptive method at last sexual intercourse	Yes	142 (53.2)	55 (50.5)	87 (55.1)	2.02 (0.365)
	No	83 (31.1)	39 (35.8)	44 (27.8)	
	Do not know	42 (15.7)	15 (13.8)	27 (17.1)	
Experience of pregnancy	Yes	12 (4.5)	5 (4.6)	7 (4.4)	0.00 (0.952)
	No	255 (95.5)	104 (95.4)	151 (95.6)	
Experience of abortion	Yes	22 (8.2)	11 (10.1)	11 (7.0)	0.84 (0.361)
	No	245 (91.8)	98 (89.9)	147 (93.0)	
Receiving Reproductive health education	Yes	2,090 (73.7)	500 (62.0)	1,590 (78.4)	79.81 (<0.001)
	No	744 (26.3)	306 (38.0)	438 (21.6)	
Receiving HIV/AIDS education	Yes	2,435 (85.9)	641 (79.5)	1,794 (88.5)	38.05 (<0.001)
	No	399 (14.1)	165 (20.5)	234 (11.5)	
Receiving contraception education	Yes	1,841 (65.0)	486 (60.3)	1,355 (66.8)	10.76 (0.001)
	No	993 (35.0)	320 (39.7)	673 (33.2)	
Knowledge of HIV/AIDS		4.0 (± 1.43)	4.06 (± 1.51)	3.91 (± 1.39)	2.41 (0.016)
Knowledge of STD	Well-known	449 (15.8)	111 (13.8)	338 (16.7)	20.58 (<0.001)
	Little known	1,509 (53.2)	396 (49.1)	1,113 (54.9)	
	No	876 (30.9)	299 (37.1)	577 (28.5)	
Knowledge of contraception	Well-known	866 (30.6)	260 (32.3)	606 (29.9)	7.53 (0.023)
	Little known	760 (26.8)	187 (23.2)	573 (28.3)	
	No	1,208 (42.6)	359 (44.5)	849 (41.9)	
Knowledge of pregnancy	Well-known	870 (30.7)	230 (28.5)	640 (31.6)	15.32 (<0.001)
	Little known	906 (32.0)	230 (28.5)	676 (33.3)	
	No	1,058 (37.3)	346 (42.9)	712 (35.1)	
Knowledge of nurture	Well-known	728 (25.7)	196 (24.3)	532 (26.2)	1.54 (0.464)
	Little known	1,199 (42.3)	354 (43.9)	845 (41.7)	
	No	907 (32.0)	256 (31.8)	651 (32.1)	
Knowledge of tuberculosis	Well-known	978 (34.5)	253 (31.4)	725 (35.7)	5.95 (0.051)
	Little known	1,111 (39.2)	341 (42.3)	770 (38.0)	
	No	745 (26.3)	212 (26.3)	533 (26.3)	
Needs of health education for students	Personal hygiene	964 (34.0)	246 (30.5)	718 (35.4)	52.52 (<0.001)
	Sexual & reproductive health	856 (30.2)	217 (26.9)	639 (31.5)	
	Healthy behaviors	309 (10.9)	77 (9.6)	232 (11.4)	
	Nutrition	154 (5.4)	42 (5.2)	112 (5.5)	
	Mental health	51 (1.8)	17 (2.1)	34 (1.7)	
	Prevention of communicable diseases	66 (2.3)	30 (3.7)	36 (1.8)	
	Do not know	434 (15.3)	177 (22.0)	257 (12.7)	

### Status of School Health in Target Areas

According to the results of key person interviews, there was no school health concept which managed health problems and prevented diseases among schoolteachers and students in target areas. The limited resources including health professional staff like school nurses, infrastructure, facilities, and health related items were related to a lack of school health concept; neither was there health education in the school curriculum. The school absence rate was reported to be high due to family problems, nutritional deficiencies, and infectious diseases, including worms, and tuberculosis. Furthermore, the school dropout rate was high due to students' pregnancies. However, there was no program, monitoring of basic data, or strategies to solve these problems.

The principals and representative teachers reported the current health problems for students were anemia, communicable diseases, tuberculosis, malnutrition, poor personal hygiene, and sexual problems. In addition, many parents did not provide care for their children because they were busy with their farm work. Regarding current health problems for teachers, the principals and representative teachers reported that stress, gastritis, hypertension, and mental health issues due to family problems were their main health problems. In terms of health-related needs for students, the principals and representative teachers reported health education about personal hygiene, reproductive health, and preventing communicable diseases as well as the school's physical environment, including lights, desk, chair, and facilities for sanitation, were needed (Table 4).

**Table 4:** Health problems and needs of health education for students and teachers.

**Categories**	**Contents**
Health problems for students	Anemia, communicable diseases including worms and tuberculosis, nutritional deficiency, poor hygiene as well as family problems such as parental indifference, sexual assault in the home, child neglect, so on, psychological problems and sexual problems
Health problems for teachers	Work related stress, lack of energy, digestive problems, gastritis, hypertension, obesity, and family problems
Needs for students	Health education including personal hygiene, nutrition, sexual and reproductive issues, and preventing communicable diseases and school physical environment including lights, desk and chair, facilities for sanitation, so on

### Components of Health Promoting School

The results of schools' current status by components of HPS found the following: Regarding the school health policy, there was no school health curriculum and no health policy in the schools. Regarding the school health service, five schools did not have a school health room or health personnel in the school. Only one school established a school health room, but there were no health personnel in the school health room. All schools had first aid kits, but most of the medical disposables were not managed. In terms of school health education, six schools had a plan of cooperation with a regional health center to provide health education; however, it was rarely implemented due to a lack of manpower and circumstances. Regarding the school environment, three schools in Perené had old and unsafe environmental conditions and the number of toilets had shortages compared to the number of students, whereas three schools in Pichanaqui had a better school building and facilities. Lastly, regarding community networks, the parents' association was not activated among the three schools in Perené while it was well-activated in the three schools in Pichanaqui. Although all six schools were linked to a local health center, the local health center supported the provision of health education to students irregularly, one to three times per semester, and conducted physical examinations for only two primary school students among six selected schools in the project area.

## Discussion

Insufficient school health policy in health education curriculum, trained teachers, and school infirmary as well as council for school health were drawn by key informants' interviews, and this finding was consistent with the survey results for teachers and students in this study. The differences in attending continuing health education for teachers resulted in their students' health status improving at the primary level and experiencing sexual intercourse at the secondary level. Teachers in the Pichanaqui area more frequently attended reproductive health education than teachers in the Perené area. This finding will result in the goal of a school health policy that prepares students for their future roles in society and prevent social inequalities through school health education about reproductive health for secondary students (18).

Those health problems and needs of students mentioned by the principals and representative teachers correlated with the major findings of the teachers' and students' surveys. However, the variance in their problems based on their enrollment in primary or secondary school was identified. The lower the school year, the more basic hygiene was an issue, whereas the higher the school year, the more sexual, and psychological problems were prominent in the students' survey results.

On the other hand, the perception of primary students in Perené showed that they rated their parents' economic status as low and their family relationships as poor, and their health status was poorer than the respondents from Pichanaqui. Those primary students in the Pichanaqui area had more compliance with hand washing than comparable students from Perené. However, their BMI scores indicating overweight and obesity were higher in the Pichanaqui area than in Perené, whereas snack and soda intake was higher in Perené. These findings regarding the difference of percentages of overweight and obesity in the pre-adolescent age group between Perené and Pichanaqui are similar to those in other studies (19, 20); BMI z-scores were high in middle- and higher-income countries, whereas low BMI scores were found in low income countries. It may explain that BMI disparity in the same province correlates with parents' income, although some studies show that there was no correlation with a BMI of overweight and physical activities (21, 22). If there was no supplementary school health program for healthy school lunches, sport, enjoyment of friends and families, and safety environment at school level (14), those inequity and disparity would be aggravated.

The health problems of secondary students in this study showed that the health behaviors of students in Perené were distinctly different regarding their receiving of reproductive health and HIV/AIDS information as well as contraception health education. They had more knowledge about HIV/AIDS despite insufficient information gathering from school and having less Internet accessibility than in Pichanaqui. This finding is consistent with previous research results that as secondary students displayed more sexually risky behaviors their HIV/AIDS knowledge could be a proxy for general reproductive health knowledge. Because they had started their sexual career earlier, these students had poorer reproductive health knowledge as well as knowledge about unwanted pregnancies (23). However, having better knowledge about HIV/AIDS does not guarantee that they have adequate knowledge; it may indicate that they sought related information more actively (18).

In terms of the health education needs of students, the same priorities between key informant interviews and survey results were found, ranging from hygiene and nutrition to sexual and reproductive health and prevention of infectious diseases by school level. Regarding mental health education, secondary students' need for it was the lowest. This finding runs counter to the high prevalence of suicidal attempts in impoverished urban areas in Peru (24). It may be explained by the distance from their residence to the main capital city takes more than 6 h by car and their way of living in a remote area is less competitive than life in an urban area.

The health problems of teachers were work-related stress, including digestive and cardiovascular problems, however, teachers had the responsibility to care for sick students because there were no school health personnel other than psychological counseling teachers. In terms of health promoting school, each component of it was not well-organized and supported by the five school principals except by one school and local health department. This particular school principal had a concept of health promoting school and endeavored that the school environment would change. However, a systematic approach based on community and parents' participation are needed to guarantee students' health, which will contribute to the healthy community members and the healthy economic growth of the future workforce in Peru.

Multiple contextual and mediating factors are associated with student health status in consideration of the outcome of school health promotion. Those factors identified in this needs assessment provided evidence about how to guarantee students' rights to health in the process of project implementation. Though the difference of health knowledge and behavior related to the economic status of their parents and their parents' involvement in school health decision-making processes, there needs to be a change regarding community participation and a collective program for healthy school environments. All these project activities should be monitored and managed by the project team in collaboration with the provincial health department to guarantee students' rights to health by strengthening school health policy with sustainability.

## Conclusion

Based on the findings from this study, multi-component school-based interventions were adjusted to increase feasibility and sustainability of the project to the two catchment areas. We adjusted the project action plan based on the five components of health promoting school. These were organizing a council for school health including parents so that they could participate in promoting a healthy environment for their children in school and at home. Another action plan was applying an annual school health plan for training teachers to provide health education to their students and the development of an illustrated manual of school health programs by school teachers especially focusing on inculcating good hygienic habits for primary students and proper decision making regarding reproductive health for secondary students. For sustainable health management in school, regular health check-up for teachers in addition to providing anthelmintic medicine to primary school students for preventing absenteeism from stomach-ache and maintaining learning time in school was suggested. All these activities were implemented throughout the project years, and pre-and post-test evaluation to compare the effect of health promoting schools will be analyzed.

However, due to lack of healthcare personnel, only one out of six schools in the catchment area had a school nurse. Thus, capacity reinforcement by monitoring the school health committee of their annual school health planning and implementation during project activities was important for project accomplishment and sustainability. Furthermore, based on positive results of the project, it was required to monitor whether the changes were implemented on the provincial school health policy through resource network among the health department, education department, and the public health center.

## Data Availability Statement

The raw data supporting the conclusions of this article will be made available by the authors, without undue reservation.

## Ethics Statement

The studies involving human participants were reviewed and approved by Chosun University IRB No. 2-1041055-AB-N-01-2019-3. Written informed consent to participate in this study was provided by the participants' legal guardian/next of kin.

## Author Contributions

YC was a major contributor in writing the manuscript and contributed to data collection. SK contributed to critical revision of the manuscript. JK, IK, and ML contributed to the conception and design of this study. All authors read and approved the final manuscript.

## Conflict of Interest

The authors declare that the research was conducted in the absence of any commercial or financial relationships that could be construed as a potential conflict of interest.
